# Warm glow feelings can promote green behavior

**DOI:** 10.1093/pnasnexus/pgae509

**Published:** 2024-11-13

**Authors:** Jennifer Jerit, Hwayong Shin, Jason Barabas

**Affiliations:** Department of Government, Dartmouth College, 3 Tuck Mall, Hanover, NH 03755, USA; Rockefeller Center for Public Policy and the Social Sciences, 6082 Rockefeller Hall, Hanover, NH 03755, USA; Department of Government, Dartmouth College, 3 Tuck Mall, Hanover, NH 03755, USA; Rockefeller Center for Public Policy and the Social Sciences, 6082 Rockefeller Hall, Hanover, NH 03755, USA

**Keywords:** proenvironmental behavior, warm glow, emotion

## Abstract

For climate mitigation to be successful, vast numbers of people must change how they go about daily life. Social scientists have tried to promote environmentally sustainable (i.e. “green”) behavior with interventions involving cues, frames, and information, but the cumulative impact of those efforts has been modest. A growing number of studies—largely observational—suggests the promise of “warm glow” messaging that features the pleasure and satisfaction one experiences from acting sustainably. While past work has established the association between intrinsic motivations and green behavior, our study offers evidence regarding the causal effect of warm glow feelings in the climate domain. In three survey experiments administered on different national samples, we induce feelings of warm glow and examine the impact on green behavioral intentions. The treatment, an adaptation of a standard feeling induction, has a significant influence on a wide range of actions—an effect that is distinct from the influence of general positivity. Most importantly, we observe the largest treatment effects in surprising places: among Republicans, and within this subgroup, on more socially visible activities. Manipulated warm glow also increases intentions to engage in more difficult (e.g. costly, effortful) activities. Our findings are valuable for scholars and practitioners seeking to promote broad-based climate mitigation across the ideological spectrum.

**Significance Statement**

Do people engage in proenvironmental (“green”) behavior because doing so makes them feel good? This question is answered across three experiments that manipulate “warm glow,” an intrinsic emotional reward that has been linked to prosocial behavior in other areas. Here, we employ a novel method for inducing warm glow feelings and observe the effects across a range of environmentally sustainable behaviors. People who experience warm glow report more green behavioral intentions than those who do not. In contrast to the observational literature on this topic, the warm glow effect is most apparent among Republicans, and within this subgroup, on more socially visible activities. Finally, warm glow is most effective at motivating more difficult (e.g. costly, effortful) forms of sustainable behavior.

## Introduction

Successful climate mitigation is going to require individual-level behavior change on a massive scale. Communication strategies that work across the political spectrum—those featuring images (1), group-based emotions (2), or other-regarding values (3)—are promising because they promote broad-based engagement with the issue. Recent work suggests that “warm glow” (WG) messaging, which highlights the pleasure and satisfaction one experiences from acting sustainably, might have similar effects (4–7). Yet nearly all the existing research on warm glow relies on observational data, which cannot identify the causal effect of warm glow feelings.

One notable exception comes from Lohmann et al. (8), who attempt to induce warm glow feelings with high production videos and measure the effect with a real-effort proenvironmental task. In this study, the authors are only partially successful at manipulating WG, and the effects of the treatment on proenvironmental behavior are null. Thus, the causal impact of warm glow in the climate domain remains unclear.

We report the results of three experiments that manipulate warm glow and observe the effects on a broad range of behavioral intentions in different national samples. We deploy a feeling induction (e.g. (9)) to exogenously induce warm glow in a nondeceptive manner. Respondents read that “Scientific studies show that taking actions to protect the environment, even small things, gives people a feeling of satisfaction,” and then are asked to “describe a time when you did something for the environment and felt good afterwards” ((10), Materials and measures section). Extensive pretesting established the effectiveness of the feeling induction compared to other methods of manipulating WG (SI Appendix).

We hypothesized that manipulated warm glow would increase a person’s intentions to engage in green behaviors relative to those who did not receive the warm glow treatment (H1).^a^ We explored three research questions suggested by the literature. Some scholars speculate that the warm glow effect might be most apparent among people who already engage in climate mitigation (e.g. Democrats/proenvironmentalists; (11)). Yet van der Linden (2018) finds the correlation between warm glow feelings and green behavior to be similar for liberals and conservatives. Accordingly, we examine whether there are heterogeneous treatment effects (HTEs) for partisan subgroups (RQ1).

The repertoire of sustainable behaviors is vast (12), so we also explore whether there are HTEs based on the *visibility* (RQ2) and the *difficulty* (RQ3) of the activity. Previous observational research finds that people take actions consistent with salient identities, resulting in a pattern whereby proenvironmentalists take high visibility actions (e.g. reusable bags) while antienvironmentalists take low visibility actions (e.g. conserve water) (13). Our second research question asks whether the effect of a warm glow treatment is conditional upon a person’s political identity and the visibility of the behavior. If that were the case, we might observe a warm glow effect for Republicans, but only on low visibility behaviors. Likewise, the treatment effect on Democrats might be especially apparent on high visibility behaviors. Finally, there is evidence that warm glow feelings are associated with low- rather than high-cost activities (14). Our third research question investigates whether treatment effects are conditional upon the difficulty of the behavior.

## Research design

To examine the proposed hypothesis and RQs, we conducted three studies: Study 1 was exploratory, and studies 2 and 3 were preregistered prior to data collection. Preregistrations are available at https://aspredicted.org/gz6q-jtnh.pdf (study 2) and https://aspredicted.org/hbwk-fdnq.pdf (study 3).

In study 1 and study 2, respondents were randomly assigned to one of two conditions (Warm Glow or Control) before answering a manipulation check and a 15-item green behavioral intentions scale (13). Analysis of the manipulation check shows the writing task elevated warm glow feelings relative to the control condition in both studies 1 and 2, but the difference is significant only in the first study ($p_{\mathrm{study}\;1}=0.043$, $p_{\mathrm{study}\;2}=0.572$).^b^

In study 3, respondents were randomly assigned to one of three conditions (Warm Glow, Placebo, Control) before completing a manipulation check and a 10-item green behavioral intentions scale (Materials and measures section). WG was manipulated in the same manner as studies 1 and 2. The placebo condition involved a writing task that was expected to be positive but unrelated to the environment (e.g. “Scientific studies show that taking up a hobby, whatever the activity, gives people a feeling of satisfaction. Please describe a time when you did something related to a hobby and felt good afterwards.”). Both the warm glow and placebo treatments are nondeceptive (10, 15), and the writing task involves self-reflection. Thus, any differences we observe in green behavior across the two conditions can be more confidently attributed to the experience of warm glow feelings, as opposed to general positivity. Analysis of the manipulation check from study 3 shows that the WG writing task elevated warm glow feelings relative to the control (p=0.027). Somewhat unexpectedly, the placebo task had a positive effect that was statistically significant (p=0.049). Structural topic models (SI Appendix) show that the content of open-ended responses differed in the expected manner: people in the warm glow condition mentioned activities related to sustainability (e.g. “trash,” “recycle,” and “plant”), while people in the Placebo condition described activities unrelated to the environment (e.g. “paint,” “read,” and “cook”).^c^

## Results

**Fig. 1.**
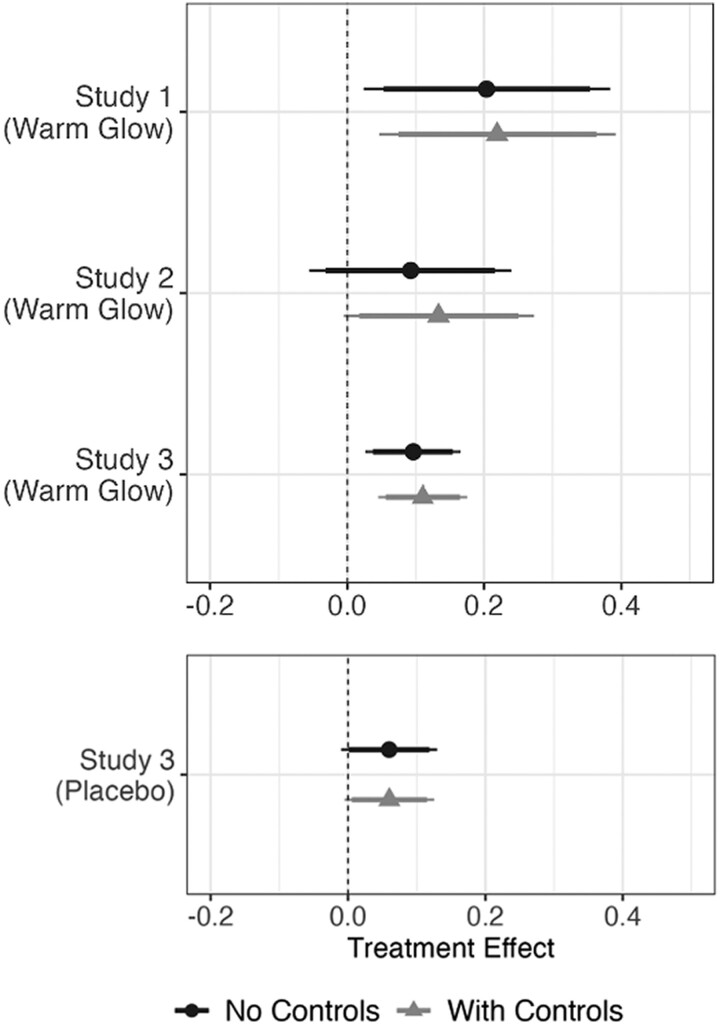
Warm glow treatment effects on Behavioral Intentions (studies 1–3). Treatment effects of warm glow induction treatment (along with 90% and 95% CI) are calculated from Ordinary Least Squares (OLS) regression models. Outcome variable is the 15-item Behavioral Intentions scale in studies 1 and 2, and the 10-item Behavioral Intentions scale in study 3 (both scales range from 1 to 7). Controls include partisanship, gender, age, education, income, Hispanic, and Black. The top panel displays the treatment effect of warm glow in study 1, study 2, and study 3. The bottom panel displays the effect of the placebo in study 3.

Figure 1 presents the treatment effects from studies 1–3 in two model specifications: a basic model with treatment identifiers as the only covariates and a model with control variables that serves as a post hoc robustness check. Across both specifications, respondents in the warm glow condition report more proenvironmental behavioral intentions than people in the control condition. The warm glow effect is significant at conventional levels in study 1 ($p_{\mathrm{no}\;\mathrm{controls}}=0.027$, $p_{\mathrm{controls}}=0.013$) and study 3 ($p_{\mathrm{no}\;\mathrm{controls}}=0.007$, $p_{\mathrm{controls}}=0.001$), and marginally significant in study 2 ($p_{\mathrm{no}\;\mathrm{controls}}=0.221$, $p_{\mathrm{controls}}=0.059$). Additionally, in study 3 there is a tendency for people in the placebo condition to report more proenvironmental behavioral intentions relative to the control ($p_{\mathrm{no}\;\mathrm{controls}}=0.093$, $p_{\mathrm{controls}}=0.070$), a finding that is consistent with previous research on the influence of positive affect on proenvironmental behavior (16).

Overall, there is a consistent pattern across the three studies in which exogenously induced warm glow feelings increase green behavioral intentions. This influence is distinct from the effect of general positivity, though the latter motivates green behavior to some degree. When it comes to the willingness to pay (WTP) for green electricity, warm glow feelings have a marginally significant effect on average contribution level ($p_{\mathrm{no}\;\mathrm{controls}}=0.208$, $p_{\mathrm{controls}}=0.081$), as does the placebo ($p_{\mathrm{no}\;\mathrm{controls}}=0.218$, $p_{\mathrm{controls}}=0.074$). As might be expected, factors like income and partisanship are strongly associated with a person’s willingness to pay for green electricity.

**Fig. 2.**
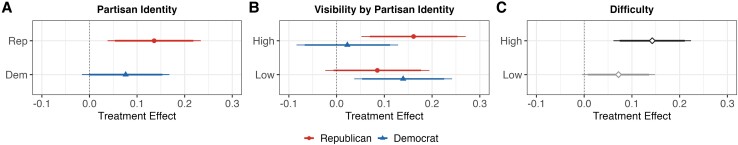
Warm Glow Treatment Effects on Behavioral Intentions by Partisan Identity and Behavior Types (study 3). Treatment effects of warm glow induction and placebo treatments (along with 90% and 95% CI) are calculated from OLS regression models where the outcome variable is the 10-item Behavioral Intentions scale (ranges from 1 to 7). A and B) Estimates for partisan subgroups are shown. C) Estimates for the entire sample are shown.

Figure 2 presents findings regarding heterogeneous treatment effects by partisanship (RQ1) as well as the visibility (RQ2) and the difficulty (RQ3) of the behavior. Past observational work suggests there may be differences in the effectiveness of a WG intervention based upon a person’s political identity and characteristics of the activity.

In Fig. 2A, the treatment effect is statistically significant for Republicans, indicating that warm glow feelings may be most influential on skeptical audiences. The partisan difference in treatment effects is not statistically significant (p=0.382), but the magnitude of the treatment effect for Republicans is almost double the size of the treatment effect for Democrats. The data reveal differences at baseline and in responsiveness to the treatment. Republicans express a significantly lower willingness to engage in sustainable activities than Democrats in the control group (t=15.86, p<0.001) and Republicans respond to a greater degree to the warm glow induction.^d^

Figure 2B investigates whether treatment effects vary with the identity-signaling potential of the behavior. One possibility, suggested by the literature (13), is that Republicans exhibit the largest treatment effects on low-visibility behaviors. As Fig. 2B shows, however, we observe larger treatment effects for Republicans than Democrats on high visibility behaviors (difference in treatment effects = 0.14, p=0.074). To the extent that Republicans prefer (and already are doing) sustainable behaviors that are less visible, warm glow feelings may motivate them to do new behaviors that are more visible.

Figure 2C explores whether there are HTEs based on the difficulty of the behavior. Past work finds that warm glow feelings are more closely associated with low- rather than high-cost activities (14). In contrast, we observe a large, statistically significant warm glow effect for high-difficulty behaviors (p=0.001) and a modest effect for low-difficulty behaviors (p=0.066).

Across the three panels, manipulated warm glow increases the willingness to take green actions in distinctive ways. The treatment is especially powerful at overcoming identity-based resistance (e.g. Republicans, particularly visible activities) and hesitation regarding high-difficulty behaviors. These findings illustrate the power of intrinsic, as opposed to extrinsic, motivations in the climate domain (18).

## Discussion

Observational research suggests the promise of the warm glow effect in providing the impetus for proenvironmental behavior (4, 5, 7, 14). Our research complements this work by identifying the causal effects of warm glow. We also advance the climate science literature in three other ways. First, we developed, validated, and documented the effectiveness of the warm glow feeling induction. The treatment is specific to a person’s own experiences, and as a result, has greater impact than previous efforts at manipulating warm glow (8). Second, through the use of a placebo condition (study 3), we showed that warm glow feelings are different from general positivity (i.e. the WG effect is intrinsic to sustainable behavior). Third, exploration of the three RQs illustrates the distinctive power of the warm glow effect. Specifically, there is a tendency for larger warm glow effects in surprising places: among Republicans, and within this subgroup, on more socially visible activities. Manipulated warm glow also increases intentions to engage in more difficult (e.g. costly, effortful) activities. These patterns contradict past observational work that finds Republicans prefer lower visibility green behaviors, and that warm glow feelings have the greatest impact on low-cost behaviors.

We can only speculate about why our findings differ from previous studies, but researchers often come to different conclusions when the focal variable is measured vs. manipulated (19, 20).^e^ One additional reason for the larger-than-expected effects on Republicans may be the language of the warm glow treatment, which asked people to write about protecting the environment (as opposed to climate change; (21)). Even though the most provocative patterns (e.g. Fig. 2) require further empirical validation, we see promise in these results for practitioners and scholars who may build upon our efforts. Warm glow messaging appears to be a potent method for targeting hard to reach audiences and motivating challenging activities. As such, this intervention makes a uniquely important contribution to broad-based climate mitigation.

At the same time, this research leaves unanswered questions, such as the duration of warm glow effects and the potential for a “virtuous cycle of positive affect” (11) as well as the differential effect of warm glow by socioeconomic status (a prospect suggested by some of the responses to the writing task). Finally, as organizations and governments innovate with green policies that include a voluntary component (22), it would be valuable to adapt the warm glow feeling induction for use in real-world messaging campaigns.

## Materials and measures

All studies received approval from the Committee for the Protection of Human Subjects at Dartmouth College: study 1 (00032816), study 2 (00032853), and study 3 (00032908). Informed consent was obtained from all participants.

The complete design of study 1 is a five-condition between-subjects experiment conducted on the CloudConnect platform in September of 2023 (N=1,646). We compared the feeling induction to three other methods of inducing warm glow and an untreated control. The feeling induction was the most effective method for inducing WG (Table S6). The text of that treatment reads: “Scientific studies show that taking actions to protect the environment, even small things, gives people a feeling of satisfaction. Please describe a time when you did something for the environment and felt good afterwards. If possible, please tell us in a few sentences and be as specific as possible. We’re interested in learning about your experience.” Analyses in the main text compare the WG feeling induction to the control condition (the other three treatment conditions are omitted).

After the treatment, respondents answered a manipulation check which asked them to rate their level of agreement with four statements (e.g. “I expect to feel good when I behave in an environmentally friendly way”) on a 7-point scale (α=0.90). The main dependent variable, separated from the treatment by six questions on unrelated topics, was a 15-item behavioral intentions battery (α=0.89) (13). That item asked respondents to rate “how likely you would be to engage in the following behaviors in the future” on an 8-point scale. The list included a variety of activities (curtailment and adoption behaviors) and had a response option for “already doing.” Values on the dependent variables represent a person’s average likelihood to engage in activities they were *not* already doing.

Study 2 was a preregistered two-condition between-subjects experiment conducted by YouGov as part of the 2023 Congressional Election Study (CES) in November of 2023 (N=1,000; AsPredicted registration #153426). The feeling induction treatment, manipulation check, and dependent variables are the same as the instrumentation in study 1 with one exception. One of the behavioral intentions changed from “Purchase clothing from environmentally friendly brands” to “Purchase clothing from environmentally friendly brands or from a thrift store” (α=0.90).

Study 3 was a preregistered three-condition between-subjects experiment conducted by Verasight in February of 2024 (N=8,323 self-reported Democrats and Republicans only; AsPredicted registration #160884). The first manipulation check, placed immediately after the treatment (23), consists of one of the four statements from the scale in study 1 (“I expect to feel good when I behave in an environmentally friendly way.”). A second manipulation check appeared after the dependent variable, and it asked respondents if while taking the survey they had read something about a certain topic, with response options for a study about “taking actions to protect the environment,” “taking up a hobby,” “sports teams around the world,” “blood donation trends,” and “did not read anything about these topics.” The primary dependent variable is similar to studies 1 and 2, but with 10 items (α=0.86). Five of the 10 questions ask about low visibility behaviors (e.g. high efficiency light bulbs), and five ask about high visibility behaviors (e.g. reusable bags). Within low/high visibility subscales, we balanced the items in terms of difficulty. We identified low/high visibility and low/high difficulty activities with a pretest from December 2023 (N=1,986) on CloudResearchConnect. In study 3, there was a WTP measure that asked respondents how much more (in dollars) they would be willing to pay for green electricity (options range from 0 to 30 dollars, with a write-in option for some other amount). Analysis of the WTP item appears in the SI Appendix.

In studies 1–3, partisanship is measured pretreatment and operationalized with a binary variable where Republican is coded as 1 and Democratic is coded as 0.

## Supplementary Material

pgae509_Supplementary_Data

## Data Availability

The study materials, data, and code for this study are available at: https://osf.io/u6rf2/ (DOI: 10.17605/OSF.IO/U6RF2).

## Notes

a Our third study includes an additional behavioral measure, willingness to pay for green electricity, which we also analyze.

b Engagement with the treatment might have differed because study 2 was longer than study 1 and included several questions on unrelated topics. Participants in study 1 wrote more in response to the prompt than those in study 2 (37.2 vs. 14.4 words). All reported statistical tests are two-tailed.

c Only a small number of people (fewer than four percent) said they had never done something for the environment or related to a hobby.

d See the SI Appendix for parallel analyses from study 1 and study 2. As scholars have observed, it is challenging to achieve sufficient statistical power to detect interactive effects (17).

e Measured WG might capture preexisting differences related to environmental attitudes, which would conflate the WG effect with those characteristics.

## Previous Presentation

These results were previously presented at the 2023 Behavioural Political Economy Workshop at London School of Economics and at the 2024 annual meetings of the Midwest Political Science Association, American Political Science Association, and the International Society of Political Psychology.
