# Supplementary material for: Warm glow feelings can promote green behavior
Source: PNAS Nexus. 2024 Nov 13;3(12):pgae509. doi: 10.1093/pnasnexus/pgae509 (PMC11631072; doi:10.1093/pnasnexus/pgae509)
Supplement: pgae509_Supplementary_Data [file pgae509_supplementary_data.pdf]

*Warm Glow Feelings Can Promote Green Behavior*

Jennifer Jerit[1], Hwayong Shin[2], Jason Barabas[1,2]

# Contents

**1 Main Analyses** **1**

1.1 Warm Glow Treatment Effects . . . . . . . . . . 1

1.2 Warm Glow Effects by Partisan Identity and Behavior Types . . . . . . . . . . 4

**2 Auxiliary Analyses** **5**

2.1 Distribution of Demographic Variables . . . . . . . . . . 5

2.2 Evidence on Treatment Effectiveness . . . . . . . . . . 6

2.3 Pilot Study for Behavior Type Validation . . . . . . . . . . 7

2.4 Power Analysis . . . . . . . . . . 8

2.5 Structural Topic Modeling Results . . . . . . . . . . 11

2.6 Additional Analyses . . . . . . . . . . 14

**3 Survey Instrumentation** **16**

3.1 Study 1 . . . . . . . . . . 16

3.2 Study 2 . . . . . . . . . . 20

3.3 Study 3 . . . . . . . . . . 21

**References** **24**

[1]Department of Government, Dartmouth College

[2]Rockefeller Center for Public Policy and the Social Sciences, Dartmouth College

# 1 Main Analyses

This section provides the analyses referenced in the main text.

## 1.1 Warm Glow Treatment Effects

Table S1 shows model output for Figure 1 in the main text (Studies 1 and 2).

Table S1: Treatment Effects on Pro-environmental Behavioral Intentions (Studies 1 and 2)

| | Study 1 | | Study 2 | |
|---|---|---|---|---|
| WG Treatment | 0.20** | 0.22** | 0.09 | 0.13* |
| | 0.09 | 0.09 | 0.08 | 0.07 |
| Democrat | | 0.57*** | | 0.48*** |
| | | 0.14 | | 0.10 |
| Republican | | −0.13 | | −0.21* |
| | | 0.15 | | 0.11 |
| Female | | −0.01 | | 0.12* |
| | | 0.09 | | 0.07 |
| Education | | 0.38** | | 0.43*** |
| | | 0.18 | | 0.13 |
| Age | | −0.01** | | −0.02*** |
| | | 0.00 | | 0.00 |
| Income | | 0.01 | | 0.08 |
| | | 0.01 | | 0.17 |
| Black | | 0.07 | | 0.10 |
| | | 0.14 | | 0.11 |
| Hispanic | | 0.19 | | −0.11 |
| | | 0.14 | | 0.11 |
| Constant | 4.23*** | 3.86*** | 4.08*** | 4.42*** |
| | 0.06 | 0.22 | 0.05 | 0.16 |
| Adj. $R^2$ | 0.01 | 0.10 | 0.00 | 0.16 |
| N | 655 | 655 | 999 | 959 |

*Note:* Cell entries are coefficients, with standard errors below, from OLS regression models where DV is the 15-item Behavioral Intentions scale. $^{***}p < .01$; $^{**}p < .05$; $^{*}p < .10$ (two-tailed).

The Behavioral Intentions columns in Table S2 provide model output for Figure 1 in the main text (Study 3).

Table S2: Treatment Effects on Pro-environmental Behavioral Intentions (Study 3)

| | Behavioral Intentions | | Willingness to Pay | |
|---|---|---|---|---|
| WG Treatment | 0.10*** | 0.11*** | 0.06 | 0.09* |
| | 0.04 | 0.03 | 0.05 | 0.05 |
| Placebo | 0.06* | 0.06* | 0.06 | 0.09* |
| | 0.04 | 0.03 | 0.05 | 0.05 |
| Republican | | −0.65*** | | −0.93*** |
| | | 0.03 | | 0.04 |
| Female | | −0.05* | | −0.01 |
| | | 0.03 | | 0.04 |
| Education | | 0.35*** | | 0.23*** |
| | | 0.04 | | 0.06 |
| Age | | −0.02*** | | −0.03*** |
| | | 0.00 | | 0.00 |
| Income | | 0.20*** | | 0.55*** |
| | | 0.05 | | 0.07 |
| Black | | 0.10** | | 0.02 |
| | | 0.05 | | 0.07 |
| Hispanic | | 0.16*** | | −0.01 |
| | | 0.04 | | 0.07 |
| Constant | 4.09*** | 4.95*** | | |
| | 0.03 | 0.06 | | |
| Coeff Test (WG=Placebo) | 0.04 | 0.05 | 0.001 | −0.002 |
| | 0.04 | 0.03 | 0.05 | 0.05 |
| Adj. $R^2$ | 0.00 | 0.14 | | |
| N | 8207 | 8092 | 8110 | 7927 |

*Note:* Cell entries are coefficients, with standard errors below, from OLS regression models where DV is the 10-item Behavioral Intentions scale and ordered logit of WTP variable (coefficients for cutpoints suppressed). $^{***}p < .01$; $^{**}p < .05$; $^{*}p < .10$ (two-tailed).

Study 3 permits an additional test of H1 with the quasi-behavioral willingness to pay (WTP) item. Respondents were asked how much more a month they would be willing to pay for green electricity (with options for $0, $10, $20, $30 or a write-in option for some other amount). Write-in responses (4%, n = 368) included a range of content—both numeric and textual.

We used `gsub` in R to extract numeric content from write-in responses. Of the 368 write-in responses, 164 indicated a dollar amounts for willingness to pay. Those responses were categorized to the following: $0 (n=2), $1-$10 (n=14), $11-$20 (n=5), $21-$30 (n=2), More than $30 (n=141). As shown in Table S3, most people who used the write-in response option indicated dollar amounts greater than $30.[1]

Table S3: Willingness to Pay (WTP): Raw and Recoded Variables

| Raw / Recoded | $0 | $10 | $20 | $30 | Some other amount (Write-in) | N |
|---|---|---|---|---|---|---|
| $0 | 3217 | 0 | 0 | 0 | 2 | 3219 |
| $1 - $10 | 0 | 1675 | 0 | 0 | 14 | 1689 |
| $11 - $20 | 0 | 0 | 1752 | 0 | 5 | 1757 |
| $21 - $30 | 0 | 0 | 0 | 1302 | 2 | 1304 |
| More than $30 | 0 | 0 | 0 | 0 | 141 | 141 |
| N | 3217 | 1675 | 1752 | 1302 | 164 | 8110 |

In Table S2, we used ordered logistic regression to analyze the effects of WG and placebo on willingness to pay. As with the patterns reported in the main text, people in the WG condition move in a pro-environmental direction compared to the control group, selecting a higher monetary contribution to green electricity ($p_{no\_controls} = .208$ and $p_{controls} = .081$). The placebo task had a marginally significant effect as well ($p_{no\_controls} = .218$ and $p_{controls} = .074$).

[1]Examples of write-in content that could not be coded to a numeric response option are entries like "tech neither C02 neutral nor clean" or "Already do and it's under 20 per month."

## 1.2 Warm Glow Effects by Partisan Identity and Behavior Types

Table S4 shows model output for Figure 2 in the main text.

Table S4: Treatment Effects by Partisanship and Behavior Types (Study 3)

| | A. Partisanship | B. Visibility | | C. Difficulty | |
|---|---|---|---|---|---|
| | | High | Low | High | Low |
| WG Treatment | 0.08 | 0.02 | 0.14*** | 0.14*** | 0.07* |
| | 0.05 | 0.05 | 0.05 | 0.04 | 0.04 |
| Placebo | 0.09* | 0.06 | 0.11** | 0.09** | 0.06 |
| | 0.05 | 0.05 | 0.05 | 0.04 | 0.04 |
| Republican | −0.75*** | −0.87*** | −0.65*** | | |
| | 0.05 | 0.05 | 0.05 | | |
| WG x Republican | 0.06 | 0.14* | −0.05 | | |
| | 0.07 | 0.08 | 0.08 | | |
| Placebo x Republican | −0.03 | 0.01 | −0.08 | | |
| | 0.07 | 0.08 | 0.08 | | |
| Constant | 4.46*** | 4.34*** | 4.60*** | 3.60*** | 4.82*** |
| | 0.03 | 0.04 | 0.04 | 0.03 | 0.03 |
| Adj. $R^2$ | 0.08 | 0.08 | 0.06 | 0.00 | 0.00 |
| N | 8206 | 8180 | 8017 | 8194 | 7646 |

*Note:* Cell entries are coefficients, with standard errors below, from OLS regression models. For panel A, DV is the 10-item Behavioral Intentions scale. For panel B, DVs are the 5-item high-visibility and the 5-item low-visibility Behavioral Intentions scale respectively. For panel C, DVs are the 5-item high-difficulty and the 5-item low-difficulty Behavioral Intentions scale respectively. $^{***}p < .01$; $^{**}p < .05$; $^{*}p < .10$ (two-tailed).

# 2 Auxiliary Analyses

## 2.1 Distribution of Demographic Variables

Table S5: Distribution of Demographics in Studies 1-3

| | Study 1 CloudConnect | Study 2 CES | Study 3 Verasight |
|---|---|---|---|
| **Gender** | | | |
| Female | 54.2 | 53.6 | 56.2 |
| Male | 43.7 | 45.8 | 43.4 |
| Other | 2.1 | 0.6 | 0.4 |
| **Partisan Identity** | | | |
| Democrat | 62.3 | 44.0 | 49.7 |
| Republican | 25.5 | 36.1 | 50.3 |
| Independent | 12.2 | 15.9 | 0.0 |
| Not sure | | 4.0 | |
| **Age** | | | |
| 18-24 | 8.2 | 9.1 | 4.9 |
| 25-34 | 27.7 | 17.3 | 17.4 |
| 35-44 | 29.2 | 14.7 | 25.9 |
| 45-54 | 16.5 | 14.1 | 21.9 |
| 55-64 | 12.4 | 20.6 | 17.9 |
| 65 or older | 6.0 | 24.2 | 11.9 |
| **Race/Ethnicity** | | | |
| White | 78.6 | 66.1 | 70.4 |
| Black | 11.7 | 13.0 | 11.3 |
| Hispanic/Latino | 10.0 | 13.5 | 11.6 |
| **Education** | | | |
| High school or less | 11.9 | 33.9 | 23.2 |
| Some college | 31.8 | 32.4 | 35.1 |
| College degree or more | 56.3 | 33.7 | 41.7 |
| **N** | 1658 | 1000 | 8323 |

*Note:* Cell entries indicate percentages of each category. For partisan identity, Independent refers to pure independents who are not partisan leaners.

## 2.2 Evidence on Treatment Effectiveness

We utilize the full design of Study 1 to test the effectiveness of different methods of inducing warm glow (WG). The key outcome in this analysis is the 4-item scale used by Jia and van der Linden (2020) to measure WG feelings. These items asked respondents to rate their level of agreement with four statements (e.g., "I expect to feel good when I behave in an environmentally friendly way") on a 7-point scale ($\alpha = .90$). If a treatment was effective at priming warm glow, respondents should have higher values on this scale. Table S6 shows the effect of the treatments on the WG manipulation check. Although coefficients for all treatments are positively signed, only the feeling induction has a statistically significant effect on WG feelings.

Table S6: Manipulation Check (Study 1)

| | Model 1 | Model 2 |
|---|---|---|
| Feeling Induction | 0.20** | 0.20** |
| | 0.10 | 0.10 |
| Choose New Action | 0.09 | 0.07 |
| | 0.10 | 0.10 |
| Scientific Study | 0.12 | 0.14 |
| | 0.10 | 0.10 |
| Third-Party Quotes | 0.15 | 0.15 |
| | 0.10 | 0.10 |
| Democrat | | 0.67*** |
| | | 0.10 |
| Republican | | 0.09 |
| | | 0.11 |
| Socially Desirable Responding (SDR) | | 0.08*** |
| | | 0.03 |
| Constant | 5.22*** | 4.75*** |
| | 0.07 | 0.11 |
| Adj. $R^2$ | 0.00 | 0.06 |
| N | 1646 | 1646 |

*Note:* Cell entries are coefficients, with standard errors below, from OLS regression models where the DV is the 4-item Warm Glow scale. Covariates are binary indicators of treatment conditions except for SDR, which is a person's score on the 8-item index rescaled to range from 0 to 1. $^{***}p < .01$; $^{**}p < .05$; $^{*}p < .10$ (two-tailed).

Compliance with the writing task was high across treatment conditions (avg length of response = 34.6 words). However, responses were longer in the Feeling Induction condition compared to the other three conditions (avg = 37.2 words; $p < .05$). In addition, people in the Feeling Induction condition were highly specific in how they talked about the environment in

their open-ended responses; for example, listing concrete actions (e.g., drying clothes outside, composting) or specific experiences (e.g., beach cleanup). These differences in respondent engagement may have resulted in a stronger effect for the feeling induction compared to the other manipulations in Study 1 as well as the treatment in Lohmann et al. (2024).

## 2.3 Pilot Study for Behavior Type Validation

We identified low/high visibility and low/high difficulty behaviors with a pre-test from December 2023 ($N = 1986$) on CloudResearchConnect. The survey asked about 23 green behaviors based on the items used in Brick et al. (2017). Participants in the pilot study rated the social visibility (how much a behavior can be observed by other people) and the difficulty (in terms of effort and expense) in two separate grids that ranged from "Not at all" (1) to "Extremely" (5). When creating the visibility subscales for Study 3, we identified items that were at the bottom and top of the visibility distribution (and significantly different from the sample mean) while balancing the item selection on difficulty.

Table S7: Selection of Items for Subscales

| Item Wording | Visibility | Difficulty |
|---|---|---|
| Limit consumption of meat and/or dairy product | 2.24 | 2.50 |
| Turn personal electronics off or put in low-power mode when not in use | 2.27 | 1.55 |
| Conserve water when showering, doing dishes, or watering plants | 2.27 | 2.13 |
| Reduce non-essential air travel | 2.32 | 2.31 |
| Use high efficiency light bulbs | 2.39 | 1.51 |
| | | |
| Use reusable bags at the grocery store | 3.58 | 1.56 |
| Walk, bicycle, carpool, or take public transportation instead of driving a vehicle | 3.61 | 3.26 |
| Carry a reusable water bottle | 3.68 | 1.40 |
| Engage in political action related to protecting the environment | 3.97 | 2.95 |
| Purchase an electric/hybrid vehicle | 4.21 | 3.81 |
| Average rating for 23 behaviours | 2.84 | 2.28 |
| | SD=0.63 | SD=0.58 |

*Note:* Visibility and Difficulty were scored on a 5-pt scale, ranging from "Not at all" (1) to "Extremely" (5). Items above the gray bar represent "low visibility" behaviors while items below it represent "high visibility" behaviors. Individual items are significantly different from the sample mean on visibility ($p$s $< .01$, two-tailed).

Among the 10 items we selected for the visibility analysis, we distinguished behaviors in terms of their difficulty. Low difficulty behaviors included: water bottle, light bulbs, turn off electronics, reusable grocery bags, and conserve water. High difficulty behaviors included: reducing air travel, limiting meat and/or dairy, political activism, alternative transportation, and EV/hybrid vehicle. The role of difficulty (i.e., RQ3) is strongly suggested by van der Linden (2018), but that analysis was not specified in the pre-registration for Study 3.

## 2.4 Power Analysis

We determined the sample size for Study 3 with power analyses, using both standard and simulation-based approaches. The purpose was to detect effects of interest and to be able to consider any potential null effects as informative as possible. We used data from Study 1 to make the assumptions about the means and standard deviations. Power analysis was based on the 8-pt pro-environmental behavioral intentions scale.

For the 15-item behavioral intentions scale, the means and standard deviations for control and treatment conditions were assumed to be: mean_control = 5.21, mean_treat = 5.41, SD_control = 1.23, SD_treat = 1.17. Table S8 summarizes the sample size per condition. Standard power analyses were conducted using `power` command in Stata. The results suggested that the sample size per condition of 601 (all respondents), 848 (Democrats), and 84 (Republicans) would achieve the statistical power of 0.80 (Figure S1).

We also conducted simulation-based power analysis. For a given sample size of $n$ and assumed treatment effects $\tau$, we conducted 1,000 simulations of data-generating process. Each simulation draws:

- $n/2$ values (number of observations under control) from a normal distribution with mean = 5.21 and SD = 1.23. These represent respondent-level PEB intentions under the control condition.
- (n - $n/2$) values from a normal distribution with mean = 5.21 + $\tau$ (assumed $\tau$ = 0.20 = 5.41 - 5.21), SD = 1.17. These represent respondent-level PEB intentions under the treatment condition.
- We conduct t-tests for the difference-in-means between control and treatment conditions and store the p-values.
- Over the 1,000 simulations for a given sample size and assumed effect, we compute power by dividing the number of simulations that produced a p-value under 0.05 by 1000.

The results of simulation-based power analysis (Figure S1, Table S8; All respondents) suggest that power of 0.80 or greater is achieved when the total sample size is 1,175 or greater (588 or greater per condition).

We also conducted simulation-based power analysis to detect meaningful heterogeneous treatment effects (HTE) by partisan groups. The means and standard deviations for control and treatment conditions were assumed to be: mean_control_dem = 5.45, mean_treat_dem = 5.61, SD_control_dem = 1.11, SD_treat_dem = 1.20 among Democrats; mean_control_rep = 4.54, mean_treat_rep = 5.09, SD_control_rep = 1.22, SD_treat_rep = 1.30 among Republicans (see Table S8 below). For a given sample size of $n$, assumed treatment effect of warm glow treatment among Democrats $\tau_{dem}$, and assumed partisan difference in treatment effects (Rep-Dem) $\tau_{rep-dem}$, we conducted 1,000 simulations of data-generating process. Each simulation draws:

- ($n_{dem}/2$) values (number of observations under control) from a normal distribution with mean = 5.45 and SD = 1.20. These represent respondent-level PEB intentions among Democrats under the control condition.

Table S8: Power Analysis: Observed Baseline Means and SD, Treatment Effects, and Proposed Sample Size

| **Study 1** | **Behavioral Intentions (15 items)** | | | |
|---|---|---|---|---|
| **All Respondents** | Mean | (SE) | (SD) | N |
| Treatment | 5.41 | 0.06 | 1.17 | 319 |
| Control | 5.21 | 0.07 | 1.23 | 336 |
| Difference (treatment effect) | 0.20 | 0.09 | | 655 |
| t-statistic | 2.07 | | | |
| p-value (two-tailed) | 0.04 | | | |
| Sample size per condition (Stata `power`) | **601** | | | |
| Sample size per condition (simulation-based) | **588** | | | |

| **Democrats** | Mean | (SE) | (SD) | N |
|---|---|---|---|---|
| Treatment | 5.61 | 0.09 | 1.11 | 162 |
| Control | 5.45 | 0.09 | 1.20 | 167 |
| Difference (treatment effect) | 0.16 | 0.12 | | 329 |
| t-test statistic | 1.24 | | | |
| p-value (two-tailed) | 0.22 | | | |
| Sample size per condition (Stata `power`) | **848** | | | |

| **Republicans** | Mean | (SE) | (SD) | N |
|---|---|---|---|---|
| Treatment | 5.09 | 0.16 | 1.30 | 69 |
| Control | 4.54 | 0.16 | 1.22 | 57 |
| Difference (treatment effect) | 0.55 | 0.23 | | 126 |
| t-test value | 2.43 | | | |
| p-value (two-tailed) | 0.02 | | | |
| Sample size per condition (Stata `power`) | **84** | | | |

| Heterogeneous Treatment Effects | Observed | Hypothetical |
|---|---|---|
| Treatment effect among Republicans | 0.55 | |
| Treatment effect among Democrats | 0.16 | |
| Partisan difference in treatment effect | 0.39 | 0.20 |
| Sample size per condition (simulation-based) | **625** | **2163** |

- $(n_{dem} - n_{dem}/2)$ values from a normal distribution with mean = 5.45 + $\tau_{dem}$ and SD = 1.11. These represent respondent-level PEB intentions among Democrats under the treatment condition.
- $(n_{rep}/2)$ values (number of observations under control) from a normal distribution with mean = 4.54 and SD = 1.22. These represent respondent-level PEB intentions among Republicans under the control condition.
- $(n_{rep} - n_{rep}/2)$ values from a normal distribution with mean = 4.54 + $\tau_{dem}$ + $\tau_{rep-dem}$ and SD = 1.30. These represent respondent-level PEB intentions among Republicans under the treatment condition.
- The simulation assumes the 5:5 ratio for the relative sample size of Democrats and

Republicans.

- We conduct t-tests for the difference-in-means of treatment effects between Democrats and Republicans and store the p-values.
- Over the 1,000 simulations for a given sample size and assumed effect, we compute power by dividing the number of simulations that produced a p-value under 0.05 by 1,000.

In Study 1, the observed difference in treatment effects between Republicans and Democrats was 0.39. Because the difference in treatment effects between partisan groups may be smaller for pro-environmental intentions subscales (e.g., high-visibility vs. low-visibility) that we intended to examine in Study 3, we also simulated data that assume a smaller difference in HTEs, 0.20 (approximately half the size of observed difference in HTEs). As shown in Figure S1, assuming $\tau_{rep-dem}$ of 0.39, power of 0.80 or greater is achieved when the total sample size is 1,250 or greater (625 or greater per condition). Assuming $\tau_{rep-dem}$ of 0.20, power of 0.80 or greater is achieved when the total sample size is 4,325 or greater (2,163 per condition).

Figure S1: Power, Effect Size, and Sample Size: 15-item Behavioral Intentions Scale

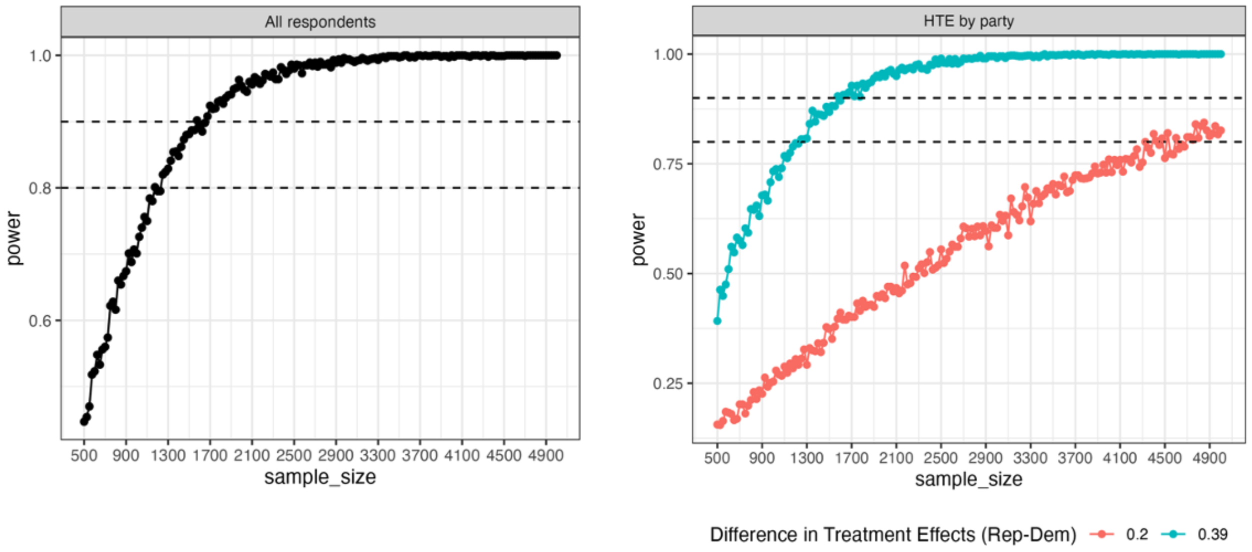


*Note:* The first dashed line (from the top) indicates .90 power, and the second dashed line indicates .80 power.

We did not conduct a power analysis for Study 2 because data collection was part of a collaborative study, with sample size fixed at n=1,000.

## 2.5 Structural Topic Modeling Results

For Study 3, we employed Structural Topic Modeling (STM) to explore respondent behavior in the WG and Placebo conditions and to further validate the effect of the WG feeling induction. Across both conditions, approximately 90% of respondents provided an open-ended response that was consistent with the instructions. A small number (less than 4%) said they could not recall a personal experience and the remainder (6-7%) left the open-ended text box blank.

Using the methods in Roberts et al. (2014) we used STMs to explore the content of the open-ended responses. The results in Figure S2 and Table S9 are based on a structural topic model that assumes 10 topics.

In Figure S2, Panel A illustrates the expected proportion of open-ended responses that belongs to each topic on the horizontal axis. It also shows three most frequent words per topic. The two most common topics are Topic 10 and Topic 7, both related to pro-environmental behaviors, followed by Topic 3, related to hobbies. Panel B compares the relative topic prevalence for each topic in WG treatment condition and placebo condition. Topics 7, 8, 10 are more prevalent among respondents assigned to WG treatment condition, whereas Topics 1-6 and 9 are more prevalent among respondents in placebo condition. Panel C illustrates correlations among topics, where positive correlations between two topics indicate that those topics are likely to be discussed within open-ended responses. The results indicate Topics 7, 8, 10 are likely to be discussed together, whereas Topics 1-6 and 9 are likely to be discussed together in open-ended responses. Lastly, Panel D lists most frequent words for each topic.

Figure S2: Structural Topic Modeling Results Assuming 10 Underlying Topics

A. Proportion of topics in the corpus (x-axis), Most frequent words for each topic

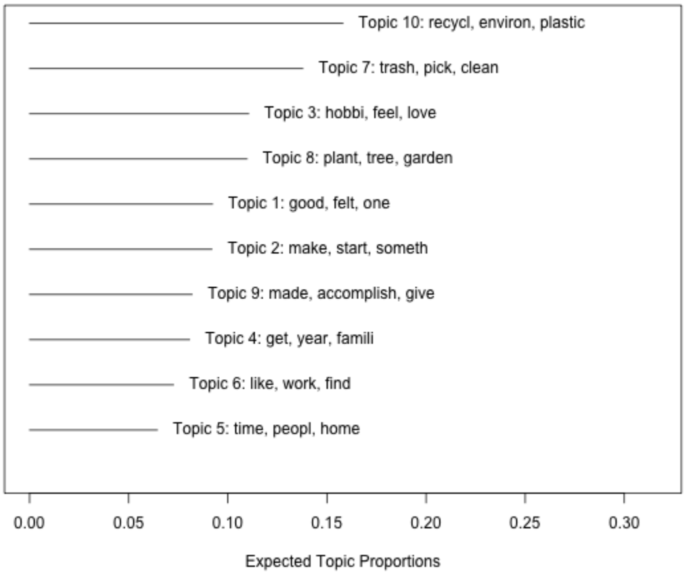


B. Topic prevalence in WG Treatment condition compared to Placebo condition

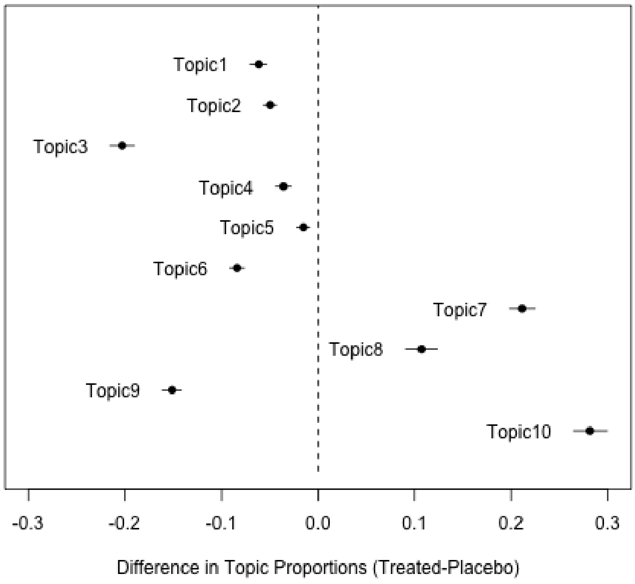


C. Topic correlations

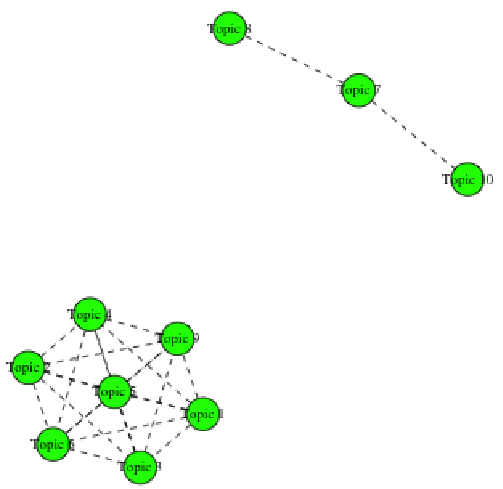

D. Frequent words for each topic

Topic 1:
good, felt, one, help, realli, enjoy, also, read, book, learn, cook, children, sport, food, other, mind, gave, free, share, keep

Topic 2:
make, start, someth, thing, take, day, happi, life, abl, just, much, now, even, first, need, know, put, iâ€™m, old, well

Topic 3:
hobbi, feel, love, play, alway, paint, great, relax, game, music, learn, enjoy, favorit, stress, draw, tri, better, exercis, write, golf

Topic 4:
get, year, famili, can, friend, took, mani, went, lot, everi, differ, walk, got, way, want, ago, fun, week, month, interest

Topic 5:
time, peopl, home, love, build, think, see, long, live, wood, outsid, piec, rememb, didnt, bring, son, small, two, built, busi

Topic 6:
like, work, find, new, fish, itâ€™, money, hous, collect, ive, sever, come, found, nice, run, recent, sinc, kid, littl, ride

Topic 7:
trash, pick, clean, use, help, water, bag, beach, park, around, road, neighborhood, walk, save, made, keep, better, along, reusabl, live

Topic 8:
plant, tree, garden, communiti, volunt, local, sens, environ, environment, particip, group, know, contribut, flower, day, posit, satisfact, grow, organ, collect

Topic 9:
made, accomplish, give, finish, satisfi, sens, enjoy, complet, creat, project, satisfact, use, joy, craft, crochet, color, art, beauti, watch, experi

Topic 10:
recycl, environ, plastic, can, tri, feel, alway, litter, garbag, thing, bottl, much, wast, also, possibl, item, throw, sure, reduc, dont

We also conducted a regression analysis in which the proportion of each topic (Topic 1 through 10) was regressed on treatment status (1 = WG; 0 = placebo), Republican (1 = Republican, 0 = Democrat), and the interaction. There is a positive and statistically significant effect for WG in models predicting Topics 7, 8, and 10 ($p < .01$; highlighted with green shading) In all other models, the coefficient for WG is negative and statistically significant.[2] These findings (similar to Panel B, Figure S2) further validate the distinctiveness of open-ended responses across the WG and Placebo conditions in Study 3.

Table S9: Associations between Topic Proportion and WG Treatment and Partisanship

| | Topic 1 | Topic 2 | Topic 3 | Topic 4 | Topic 5 |
|---|---|---|---|---|---|
| WG Treatment | −0.061*** | −0.049*** | −0.203*** | −0.036*** | −0.015*** |
| Republican | −0.010** | 0.013*** | −0.015*** | 0.002 | 0.014*** |
| WG x Republican | −0.012* | −0.018*** | 0.013* | −0.002 | −0.017*** |
| Constant | 0.130*** | 0.110*** | 0.214*** | 0.098*** | 0.072*** |
| | Topic 6 | Topic 7 | Topic 8 | Topic 9 | Topic 10 |
| WG Treatment | −0.084*** | 0.211*** | 0.107*** | −0.151*** | 0.281*** |
| Republican | −0.002 | −0.0004 | −0.004 | 0.006 | −0.003 |
| WG x Republican | 0.013** | 0.033*** | −0.007 | −0.007 | 0.005 |
| Constant | 0.114*** | 0.019*** | 0.074*** | 0.157*** | 0.012** |

*Note:* Cell entries are coefficients, from OLS regression models where DV is the proportion of each topic in open-ended responses. ***$p < .01$; **$p < .05$; *$p < .10$ (two-tailed).

[2] STM, like other probabilistic topic modeling techniques, can generate slightly different results across iterations even with the same seed. Substantive findings remain consistent across different iterations.

## 2.6 Additional Analyses

Table S10: Heterogeneous Treatment Effects by Partisan Identity and Environmental Identity (Studies 1 and 2)

| | Pro-environmental Behavioral Intentions | | |
|---|---|---|---|
| | Study 1 | Study 2 | Study 2 |
| WG Treatment | 0.11 | 0.03 | 0.12 |
| | 0.11 | 0.11 | 0.10 |
| Republican | $-1.03^{***}$ | $-0.88^{***}$ | |
| | 0.15 | 0.11 | |
| WG x Republican | $0.56^{***}$ | 0.15 | |
| | 0.20 | 0.16 | |
| Environmental Identity | | | $1.09^{***}$ |
| | | | 0.10 |
| WG x Environmental Identity | | | $-0.01$ |
| | | | 0.14 |
| Constant | $4.54^{***}$ | $4.50^{***}$ | $3.44^{***}$ |
| | 0.08 | 0.08 | 0.07 |
| Adj. $R^2$ | 0.10 | 0.11 | 0.20 |
| N | 577 | 800 | 999 |

*Note:* Cell entries are coefficients, with standard errors below, from OLS regression models where DV is 15-item Behavioral Intentions scale. Environmental identity is constructed as the median split. $^{***}p < .01$; $^{**}p < .05$; $^{*}p < .10$ (two-tailed).

Paralleling the analyses reported in the main text, we investigated whether there were heterogeneous treatment effects by partisan identity (Study 1, Study 2) and environmental identity (Study 2). As indicated by the coefficients for the interaction terms, the partisan difference in treatment effects is statistically significant in Study 1 but not Study 2. Environmental identity was positively correlated with green behavioral intentions, but the difference in treatment effects for respondents with strong versus weak environmental identity was not statistically significant.

Table S11: Willingness to Pay and Policy Results (Study 3)

| | WTP | | Policy Role | | Federal Policy | |
|---|---|---|---|---|---|---|
| WG Treatment | 0.06 | 0.09* | 0.05 | 0.08** | 0.02 | 0.04 |
| | 0.05 | 0.05 | 0.03 | 0.03 | 0.03 | 0.03 |
| Placebo | 0.06 | 0.09* | 0.00 | 0.02 | −0.02 | 0.00 |
| | 0.05 | 0.05 | 0.03 | 0.03 | 0.03 | 0.03 |
| Republican | | −0.93*** | | −0.88*** | | −0.86*** |
| | | 0.04 | | 0.03 | | 0.02 |
| Female | | −0.01 | | −0.23*** | | −0.16*** |
| | | 0.04 | | 0.03 | | 0.02 |
| Education | | 0.23*** | | 0.01 | | 0.20*** |
| | | 0.06 | | 0.04 | | 0.03 |
| Age | | −0.03*** | | −0.01*** | | 0.00*** |
| | | 0.00 | | 0.00 | | 0.00 |
| Income | | 0.55*** | | −0.13*** | | −0.03 |
| | | 0.07 | | 0.04 | | 0.04 |
| Black | | 0.02 | | −0.19*** | | −0.18*** |
| | | 0.07 | | 0.04 | | 0.04 |
| Hispanic | | −0.02 | | −0.04 | | −0.09*** |
| | | 0.07 | | 0.04 | | 0.03 |
| Constant | | | 3.88*** | 4.74*** | 3.65*** | 4.16*** |
| | | | 0.02 | 0.05 | 0.02 | 0.05 |
| N | 8110 | 7927 | 8317 | 8132 | 8250 | 8137 |
| Adj. $R^2$ | | | 0.00 | 0.15 | 0.00 | 0.18 |

*Note:* Cell entries are coefficients, with standard errors below, from OLS regression models where DV is Cell entries are coefficients, with standard errors below, from ordered logit of WTP variable (coefficients for cutpoints suppressed) and OLS regression models where DVs are Policy Role and Federal Policy. Question wording appears in Section 3.3.
$^{***}p < .01$; $^{**}p < .05$; $^{*}p < .10$ (two-tailed).

Table S11 reports the results of analyses that were preregistered but not featured in the main text. In the WTP model (also shown in Table S2), WG feelings have a marginally significant effect on average contribution level, but only in the model with covariates. As might be expected, factors like income, age, and especially partisanship are associated with a person's response to the WTP question. The next two sets of models show that partisanship and demographic characteristics have a stronger relationship with policy preferences than WG feelings. When it comes to respondents' preferences for more or less government involvement in protecting the environment, the WG treatment has a significant effect, but only in the model with covariates ($p = .10$ in the basic model without covariates). WG feelings did not have a significant effect on preferences regarding specific federal policies.

Table S12: Manipulation Check (Study 3)

| | WG Manipulation Check | Placebo Manipulation Check |
|---|---|---|
| WG Treatment | 0.39*** | −0.11 |
| | 0.06 | 0.12 |
| Placebo | −0.66*** | 1.97*** |
| | 0.05 | 0.09 |
| Constant | 0.53*** | −2.82*** |
| | 0.04 | 0.08 |
| N | 8315 | 8315 |

*Note:* Cell entries are coefficients, with standard errors below, from ordered logit of manipulation check variables (*WG manipulation check*: 1 = read about taking actions to protect environment, 0 otherwise; *Placebo manipulation check*: 1 = read about taking up a hobby, 0 otherwise; coefficients for cutpoints suppressed). ***$p < .01$; **$p < .05$; *$p < .10$ (two-tailed).

Table S12 shows that treatment assignment strongly predicted responses to the second manipulation check item, which appeared after the dependent variable. This item asked people whether they had "read something about" a certain topic, with response options for "taking actions to protect the environment," "taking up a hobby," "sports teams around the world," "blood donation trends," and "did not read anything about these topics."

# 3 Survey Instrumentation

Here we present the wording of the treatments and key variables. See codebooks accompanying replication materials for additional documentation.

## 3.1 Study 1

The first treatment (Feeling Induction) is based on the induction method used in emotion research (Banks and Valentino 2012). The second treatment (Choose New Action) is adapted from the content-controlled method for measuring tolerance (Sullivan et al. 1982). The third treatment (Scientific Study) describes the findings from a scientific study about the relationship between pro-environmental behaviors and life satisfaction and asks respondents to speculate about the reason for that finding (as in Groenendyk and Krupnikov 2021). The fourth treatment (Third-Party Quotes) invokes the above relationship but adds quotations from interviewees in the scientific study (adapting the vignette approach of Carlson and Settle 2022).

All the claims in Treatments 1-4 are non-deceptive. Past research has shown an association between pro-environmental behaviors and life satisfaction (e.g., Schmitt et al. 2018).

**Treatment Wording**

For all treatments, the text box appears on the same screen as the treatment.

**TREATMENT 1 (Feeling Induction)**

Scientific studies show that taking actions to protect the environment, even small things, gives people a feeling of satisfaction. Please describe a time when you did something for the environment and felt good afterwards. If possible, please tell us in a few sentences and be as specific as possible. We're interested in learning about your experience.

**TREATMENT 2 (Choose New Action)**

Many people try to do something, however small, to help the planet. They also think about additional ways they could contribute to climate protection in the future.

Please look at the list below. Is there anything you are not currently doing for the environment that you think you might start doing? If you see more than one idea you like, pick the one that best fits your lifestyle.

change your diet
take fewer flights
eat organic or local food
buy a more efficient vehicle
reduce use of gas-powered vehicle
cut energy use in home
use public transportation
buy items secondhand instead of new
conserve water
reduce food waste

If you were able to do [PIPE IN RESPONSE], how do you think it would make you feel? Please give us your thoughts in a few sentences.

**TREATMENT 3 (Scientific Study)**

According to a recent scientific study of citizens in the U.S. and Canada, people who engaged in more pro-environmental behaviors reported higher life satisfaction than those who did fewer. Similar results were found in research from Sweden, Mexico, Spain, and China. All around the world, contributing to the health of the planet increases feelings of personal well-being.

If you had to explain why pro-environmental behavior is linked to life satisfaction, what would you say? Please share your thoughts in a few sentences and be as specific as possible.

**TREATMENT 4 (Third-Party Quotes)**

A recent scientific study shows that taking actions to protect the environment gives people a feeling of satisfaction. Here are some of the reactions from people in this study:[3]

- ◇ Female, 55: I just think that I am doing something. I can't be a member of Greenpeace or anything like that. I know it's very small. But it's positive; it's something I feel good about.
- ◇ Male, 40: I feel like that there is a small part of me that is making a bit of a difference. Probably not a huge amount in the overall scheme of things but I feel better in my mind and my heart.
- ◇ Female, 54: I feel good that I am doing what I can. There is a good feeling that at least I am doing something.
- ◇ Male, 43: It mainly comes down to that feeling of pride and satisfaction in doing something that helps, while knowing it's never going to be quite enough, it does feel good to know that I can do something. I'm happy that I can do something.

Now imagine that *you* did something beneficial for the environment. How do you think it would make you feel? Please give us your thoughts in a few sentences and be as specific as possible.

## Question Wording

*Manipulation Check*

Please read the statements below and indicate your level of agreement or disagreement with them.

"I expect to feel good when I behave in an environmentally friendly way."
"I anticipate that I would feel good when I do something to help the environment."
"I'd feel guilty if I did NOT behave in an environmentally friendly way."
"Doing something good for the environment would make me feel positive about myself."

1. Strongly agree
2. Agree
3. Slightly agree
4. Neither agree nor disagree
5. Slightly disagree
6. Disagree
7. Strongly disagree

[3]The quotes are excerpts from interviewees in Hartmann et al. (2017, see Appendix 2)

*Behavioral Intentions Scale*

Please read the list below and tell us how likely you would be to engage in the following behaviors in the future. Don't feel any pressure, just indicate what you are likely to do.

Use reusable bags at the grocery store
Walk, bicycle, or take public transportation instead of driving a vehicle by yourself
Limit non-essential air travel
Compost your household food garbage
Limit consumption of meat and dairy products
Eat organic/locally produced food
Purchase an electric or hybrid vehicle
Install energy efficient appliances in your home
Turn personal electronics off or in low-power mode when not in use
Buy high efficiency light bulbs
Conserve water when showering, doing dishes, or watering plants
Dry clothes on a clothesline instead of using the dryer
Purchase clothing from environmentally friendly brands
Carry a reusable water bottle
Engage in political action related to protecting the environment

1. Extremely unlikely
2. Very unlikely
3. Somewhat unlikely
4. Neither unlikely nor likely
5. Somewhat likely
6. Very likely
7. Extremely likely
8. Already doing

*Socially Desirable Responding Scale*

Moving on to a different topic, how much do you agree with the following statements?

I sometimes tell lies if I have to.
I never cover up my mistakes.
There have been occasions when I have taken advantage of someone.
I sometimes try to get even rather than forgive and forget.
I have said something bad about a friend behind his or her back.

1. Strongly disagree
2. Disagree
3. Slightly disagree
4. Neither disagree nor agree
5. Slightly agree
6. Agree
7. Strongly agree

### 3.2 Study 2

**Treatment Wording**

The text box appears on the same screen as the treatment.

FEELING INDUCTION

> Scientific studies show that taking actions to protect the environment, even small things, gives people a feeling of satisfaction. Please describe a time when you did something for the environment and felt good afterwards. If possible, please tell us in a few sentences and be as specific as possible. We're interested in learning about your experience.

**Question Wording**

*Manipulation Check 1*

Please read the statements below and indicate your level of agreement or disagreement with them.

"I expect to feel good when I behave in an environmentally friendly way."

"I anticipate that I would feel good when I do something to help the environment."

"I'd feel guilty if I did NOT behave in an environmentally friendly way."

"Doing something good for the environment would make me feel positive about myself."

"I don't think I would feel any different if I did something to help the environment." (*Reverse-coded*)

1. Strongly agree
2. Agree
3. Slightly agree
4. Neither agree nor disagree
5. Slightly disagree
6. Disagree
7. Strongly disagree

*Behavioral Intentions Scale*

Please read the list below and tell us how likely you would be to engage in the following behaviors in the future. Don't feel any pressure, just indicate what you are likely to do.

Use reusable bags at the grocery store
Walk, bicycle, or take public transportation instead of driving a vehicle by yourself
Limit non-essential air travel
Compost your household food garbage

Limit consumption of meat and dairy products
Eat organic/locally produced food
Purchase an electric or hybrid vehicle
Install energy efficient appliances in your home
Turn personal electronics off or in low-power mode when not in use
Buy high efficiency light bulbs
Conserve water when showering, doing dishes, or watering plants
Dry clothes on a clothesline instead of using the dryer
Purchase clothing from environmentally friendly brands or from a thrift store
Carry a reusable water bottle
Engage in political action related to protecting the environment

1. Extremely unlikely
2. Very unlikely
3. Somewhat unlikely
4. Neither unlikely nor likely
5. Somewhat likely
6. Very likely
7. Extremely likely
8. Already doing

## 3.3 Study 3

**Treatment Wording**

For all treatments, the text box appears on the same screen as the treatment.

FEELING INDUCTION

> Scientific studies show that taking actions to protect the environment, even small things, gives people a feeling of satisfaction. Please describe a time when you did something for the environment and felt good afterwards. If possible, please tell us in a few sentences and be as specific as possible. We're interested in learning about your experience.

PLACEBO

> Scientific studies show that taking up a hobby, whatever the activity, gives people a feeling of satisfaction. Please describe a time when you did something related to a hobby and felt good afterwards. If possible, please tell us in a few sentences and be as specific as possible. We're interested in learning about your experience.

## Question Wording

*Manipulation Check*

How much do you agree or disagree with the following statement:

"I expect to feel good when I behave in an environmentally friendly way."

1. Strongly agree
2. Agree
3. Slightly agree
4. Neither agree nor disagree
5. Slightly disagree
6. Disagree
7. Strongly disagree

*Behavioral Intentions Scale*[4]

Please read the list below and tell us how likely you would be to engage in the following behaviors in the future (e.g., the next year). Don't feel any pressure, just indicate what you are likely to do.

Turn personal electronics off or in low-power mode when not in use
Use high efficiency light bulbs
Limit consumption of meat and/or dairy products
Reduce non-essential air travel
Conserve water when showering, doing dishes, or watering plants
Use reusable bags at the grocery store
Engage in political action related to protecting the environment
Carry a reusable water bottle
Walk, bicycle, or take public transportation instead of driving a vehicle by yourself
Purchase an electric or hybrid vehicle

1. Extremely unlikely
2. Very unlikely
3. Somewhat unlikely
4. Neither unlikely nor likely
5. Somewhat likely
6. Very likely
7. Extremely likely
8. Already doing

[4]In the question used for Study 3, the top 5 items are the low visibility behaviors and the bottom 5 items are high visibility behaviors. In the administration of Study 3, the order of behaviors was randomized.

*Manipulation Check 2*

We want to make sure that parts of this survey were clear to you. Please indicate if you read something about any of the following:

1. A study about taking actions to protect the environment
2. A study about taking up a hobby
3. A report about sports teams around the world
4. A report about blood donation trends
5. I did not read anything about these topics

*Policy Role*

Please tell us your opinion about the role of the federal and state governments in protecting the environment. Should the government be doing...

1. A lot less
2. Somewhat less
3. The same that it is now
4. Somewhat more
5. A lot more
6. Haven't thought much about it

*Federal Policy Grid*

Do you favor or oppose the following federal policies to reduce the effects of global climate change?

Taxing corporations based on the amount of carbon emissions they produce
Providing a tax credit to businesses that develop carbon capture technologies
Stricter restrictions on carbon emissions from power plants
Stricture fuel efficiency standards for automobiles and trucks

1. Strongly support
2. Somewhat support
3. Neutral
4. Somewhat oppose
5. Strongly oppose

*Willingness to Pay*

If you had to decide about a new electricity contract for your home, how much more (in dollars) would you be willing to pay each month for green electricity (e.g., solar, wind, geothermal) instead of electricity from non-renewable sources.

1. $0 (nothing)
2. $10
3. $20
4. $30
5. Some other amount: ___________

# References

Antoine J. Banks and Nicholas A. Valentino. Emotional substrates of white racial attitudes. *American Journal of Political Science*, 56(2):286–297, 2012.

Cameron Brick, David K. Sherman, and Heejung S. Kim. "Green to be seen" and "brown to keep down": Visibility moderates the effect of identity on pro-environmental behavior. *Journal of Environmental Psychology*, 51:226–238, August 2017. ISSN 0272-4944.

Taylor N. Carlson and Jaime E. Settle. *What goes without saying: Navigating political discussion in America*. Cambridge University Press, Cambridge, 2022.

Eric Groenendyk and Yanna Krupnikov. What motivates reasoning? A theory of goal-dependent political evaluation. *American Journal of Political Science*, 65(1), 2021.

Patrick Hartmann, Martin Eisend, Vanessa Apaolaza, and Clare D'Souza. Warm glow vs. altruistic values: How important is intrinsic emotional reward in proenvironmental behavior? *Journal of Environmental Psychology*, 52:43–55, October 2017. ISSN 0272-4944. doi: 10.1016/j.jenvp.2017.05.006.

Lili Jia and Sander van der Linden. Green but not altruistic warm-glow predicts conservation behavior. *Conservation Science and Practice*, 2(7):e211, 2020. ISSN 2578-4854. doi: 10.1111/csp2.211.

Paul M. Lohmann, Elisabeth Gsottbauer, Sander van der Linden, and Andreas Kontoleon. Chilling results: How explicit warm glow appeals fail to boost pro-environmental behaviour. *Behavioural Public Policy*, pages 1–26, March 2024. ISSN 2398-063X, 2398-0648. doi: 10.1017/bpp.2024.4.

Margaret E. Roberts, Brandon M. Stewart, Dustin Tingley, Christopher Lucas, Jetson Leder-Luis, Shana Kushner Gadarian, Bethany Albertson, and David G. Rand. Structural topic models for open-ended survey responses. *American Journal of Political Science*, 58(4): 1064–1082, 2014. ISSN 1540-5907.

Michael T. Schmitt, Lara B. Aknin, Jonn Axsen, and Rachael L. Shwom. Unpacking the relationships between pro-environmental behavior, life satisfaction, and perceived ecological threat. *Ecological Economics*, 143:130–140, January 2018. ISSN 0921-8009. doi: 10.1016/j.ecolecon.2017.07.007.

John L. Sullivan, James Piereson, and George E. Marcus. *Political tolerance and American democracy*. University of Chicago Press, Chicago, IL, May 1982. ISBN 978-0-226-77992-8. URL `https://press.uchicago.edu/ucp/books/book/chicago/P/bo3775122.html`.

Sander van der Linden. Warm glow is associated with low- but not high-cost sustainable behaviour. *Nature Sustainability*, 1(1):28–30, January 2018. ISSN 2398-9629. doi: 10.1038/s41893-017-0001-0. Publisher: Nature Publishing Group.
